# Beyond Glucose: Palmitic Acid Influences VEGFA-VEGFR2 Angiogenic Signaling in Müller Glial Cells

**DOI:** 10.3390/ijms27115144

**Published:** 2026-06-05

**Authors:** Jesus Silvestre Albert-Garay, Alan E. Medina Arellano, Karla Hernández-Fonseca, Tania Medina-Sánchez, Matilde Ruiz-Cruz, Lenin Ochoa-de la Paz

**Affiliations:** 1 Laboratorio de Neurobiología Molecular y Celular de la Glía, Departamento de Bioquímica, Facultad de Medicina, Universidad Nacional Autónoma de México, Mexico City C.P. 04510, Mexico; silvestre.alb@gmail.com (J.S.A.-G.); aemmarell@gmail.com (A.E.M.A.); 2 Unidad de Investigación, Asociación Para Evitar la Ceguera en México I.A.P., Mexico City C.P. 04030, Mexico; matilde.ruiz@apec.com.mx; 3 Laboratorio de Neuroquímica, Subdirección de Investigaciones Clínicas, Instituto Nacional de Psiquiatría “Ramón de la Fuente Muñiz”, Mexico City C.P. 14370, Mexico; karla.lapin@gmail.com (K.H.-F.); taammss@hotmail.com (T.M.-S.)

**Keywords:** Müller glial cell, palmitic acid, VEGFA, VEGFR2, diabetic retinopathy

## Abstract

Research on diabetic retinopathy (DR) usually emphasizes hyperglycemia and other causes like dyslipidemia, which are still not well understood. This study examined the effects of palmitic acid (PA) exposure, alone and combined with high glucose (G25), on Müller Glial Cell (MGC) dysfunction and angiogenic signaling. Primary MGC cultures were treated with G25 (25 mM), PA (250 µM), or PA + G25 for 24 and 48 h, followed by assessments of cell viability and analysis of the Vascular Endothelial Growth Factor (VEGFA)/VEGFA receptor 2 (VEGFR2) pathway through immunofluorescence, Western blot, and ELISA. Additionally, Gaussian mixture models (GMMs) were used to identify phenotypic subpopulations based on fluorescence intensity. The results showed that while hyperglycemia did not cause significant changes, PA and PA + G25 induced apoptosis-related cell death and significantly increased the expression of VEGFA, VEGFR2, HIF-α, and SP1. Although broad phenotypic diversity was observed at 24 h, by 48 h, a distinct shift towards an angiogenic phenotype was noted, with significantly elevated VEGFA/VEGFR2 levels. In summary, this research demonstrates that PA acts as a critical inducer of an angiogenic secretory phenotype in MGCs, indicating that lipid-mediated signaling plays a vital role in neovascularization in DR, possibly independent of glucose levels.

## 1. Introduction

Diabetes mellitus (DM) is a chronic metabolic disorder characterized by persistent hyperglycemia caused by impaired insulin secretion or insulin resistance. Type 2 diabetes (T2DM) remains the most common form, accounting for over 90% of cases worldwide, and is closely associated with the growing obesity epidemic [1]. In addition to hyperglycemia, T2DM patients often experience dyslipidemia, marked by elevated triglycerides and free fatty acids (FFAs), along with decreased high-density lipoprotein (HDL) levels [2,3]. Several studies indicate that elevated FFA levels are a key factor in insulin resistance related to T2DM, although the exact mechanisms behind this association are not fully understood [4,5]. Among these, palmitic acid (PA) is the most abundant saturated fatty acid in human circulation. In vitro studies have shown that exposure to PA causes lipotoxicity at the cellular level through activation of inflammatory pathways, endoplasmic reticulum stress, and mitochondrial dysfunction [6].

Diabetic retinopathy (DR) is a microvascular complication of DM and a leading cause of vision loss in the working-age population [7,8]. Clinically, DR progresses from non-proliferative diabetic retinopathy (NPDR), characterized by microaneurysms and hemorrhages, to proliferative diabetic retinopathy (PDR), marked by abnormal neovascularization [7]. While vascular changes are the main clinical features of DR, early stages also involve significant alterations in neuroglia. Müller glial cells (MGCs) are the primary macroglia in the mammalian retina. MGCs support retinal health by regulating synaptic activity, providing metabolic support, and helping maintain the integrity of the inner blood-retinal barrier (iBRB) [8,9]. In animal models and in patients with diabetes, MGCs undergo reactive gliosis, as shown by GFAP overexpression, cellular hypertrophy, increased production of reactive oxygen species, and the release of pro-inflammatory and angiogenic factors [10,11].

A key factor in PDR progression is the overproduction of vascular endothelial growth factor-A (VEGFA). VEGFA encourages angiogenesis by binding to its receptors, VEGFR2; receptor activation in endothelial cells triggers signaling pathways that stimulate cell proliferation and migration [7]. Elevated VEGFA levels have been observed in the vitreous humor of diabetic patients, and the primary treatment for controlling neovascularization involves VEGFA antibodies. However, these therapies often offer only temporary relief and do not address the underlying metabolic stressors associated with the disease [8,9]. Although hyperglycemia has been widely studied, the specific role of hyperlipidemia in MGCs remains insufficiently understood. Therefore, this study aims to examine the individual and combined effects of PA and hyperglycemia on the VEGFA/VEGFR2 pathway in MGCs. We employ probabilistic modeling to uncover how these metabolic stressors differentially influence phenotypic heterogeneity and angiogenic signaling, offering new insights into the complex molecular mechanisms of glucolipotoxicity in diabetic retinopathy.

## 2. Results

### 2.1. Exposure to Palmitic Acid Triggers a Cytotoxic Effect in MGCs

**Figure 1:**
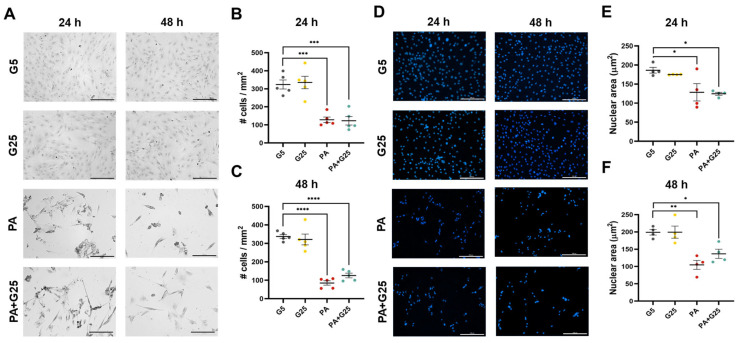
Palmitic acid decreases cell density and causes nuclear condensation in MGCs: (**A**) Representative brightfield images of MGCs treated with control (G5), high glucose (G25), palmitic acid (PA), and the combination (PA + G25). Quantification of cell density (cells/mm^2^) at 24 h (**B**) and 48 h (**C**). Data are shown as mean ± SEM of counts from three random fields per well across five independent experiments (*n* = 5). (**D**) Representative fluorescence images of DAPI-stained nuclei (blue) showing morphological changes. Quantification of nuclear area (μm^2^) at 24 h (**E**) and 48 h (**F**). Data are presented as mean ± SEM of 100 nuclei analyzed from four independent experiments (*n* = 4). Scale bars: 200 µm. Statistical significance relative to the G5 control is indicated as * *p* < 0.05, ** *p* < 0.01, *** *p* < 0.001, and **** *p* < 0.0001.

To evaluate the impact of metabolic stress on MGC viability, we first measured cell density and nuclear size of MGCs under conditions of normal glucose (G5; 5 mM), high glucose (G25; 25 mM), palmitic acid (PA; 250 μM), and the combined application of high glucose and palmitic acid (PA + G25) for 24 and 48 h. Exposure to G25 did not alter cell density at any time point examined. In contrast, treatment with PA and PA + G25 resulted in a significant reduction in cell density from 24 to 48 h (Figure 1A–C). This was also accompanied by nuclear condensation (Figure 1D–F). 

**Figure 2:**
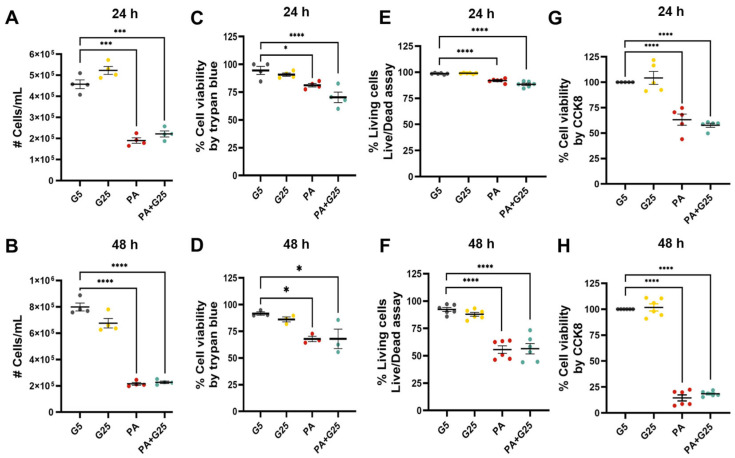
Palmitic acid decreases cell viability in MGCs. Cells were exposed to normal glucose (G5), high glucose (G25), palmitic acid (PA), and the combination (PA + G25) for 24 and 48 h. (**A**,**B**) Total cell number (#Cells/mL; *n* = 4) was quantified. (**C**,**D**) The percentage of viable cells was measured using the trypan blue exclusion assay (*n* = 4). (**E**,**F**) The percentage of live cells positive for Calcein-AM and negative for Ethidium homodimer staining was determined (*n* = 6). (**G**,**H**) Mitochondrial metabolic activity was evaluated with the CCK-8 assay (% cell viability; *n* = 5). Data are presented as mean ± SEM. Statistical significance compared to the G5 control is indicated as * *p* < 0.05, *** *p* < 0.001, and **** *p* < 0.0001.

To further characterize the cytotoxic effects of PA, we assessed plasma membrane integrity using Trypan Blue exclusion and Live/Dead assays, and mitochondrial metabolic activity with the CCK8 assay. Consistent with our initial findings, PA and PA + G25 significantly decreased total cell counts (Figure 2A,B), the percentage of viable cells (Figure 2C–F), and metabolic activity (Figure 2G,H) at both 24 and 48 h. Notably, the magnitude of the decline in cell viability varied across assays. The cell density and CCK8 assays showed a more pronounced reduction than the Trypan Blue and Live/Dead assays. This difference reflects the state of the cell culture: the Trypan Blue and Live/Dead assays primarily show the viability of the MGCs that remain attached to the plate (survivors). In contrast, the significant drop in total cell density and metabolic activity shows how the overall cell population decreases compared to the control (G5). Notably, neither high glucose levels (Figure 2A–H) nor the osmotic control mannitol (20 mM) (Figure S1) impacted cell viability. These results indicate that the effects observed in the PA + G25 group are mainly due to PA rather than to hyperglycemia-induced cytotoxicity. 

### 2.2. Temporal Dynamics and Heterogeneity of VEGFA Expression in MGCs Exposed to Hyperglycemia/Hyperlipidemia

**Figure 3:**
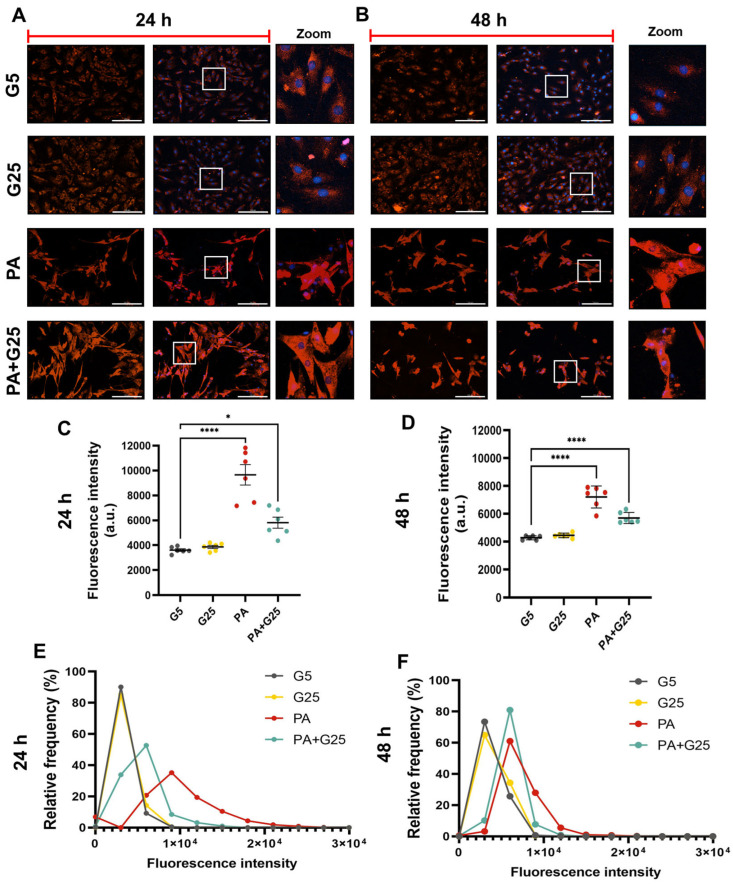
Palmitic acid causes variable VEGFA expression in MGCs: (**A**,**B**) Representative immunofluorescence images showing VEGFA (red) and nuclei (DAPI, blue) in cells exposed to the indicated conditions for 24 and 48 h. (**C**,**D**) Quantification of mean fluorescence intensity (arbitrary units, a.u.). Data are presented as mean ± SEM from six independent experiments (100 cells analyzed per experiment; *n* = 6). (**E**,**F**) Relative frequency distribution (%) of fluorescence intensity per cell (total pooled *n* = 600 cells). Cells were treated with normal glucose (G5), high glucose (G25), palmitic acid (PA), and the combination (PA + G25) at 24 and 48 h. Scale bars: 200 µm. Statistical significance compared to the G5 control is indicated as * *p* < 0.05 and **** *p* < 0.0001. (**C**,**D**) Significant differences were noted between the PA and PA + G25 groups (*p* < 0.0001) at both time points.

Following cytotoxicity assessment, we analyzed VEGFA expression and secretion in MGCs. Under G5 and G25 conditions, MGCs showed VEGFA immunoreactivity, primarily perinuclear with diffuse cytoplasmic staining (Figure 3A,B). G25 exposure did not significantly alter VEGFA levels or localization compared to controls. In contrast, PA and PA + G25 significantly increased VEGFA expression (Figure 3A,B). Quantitative analysis confirmed a substantial rise in mean fluorescence intensity in PA-treated cells compared to controls at 24 h (Figure 3C) and 48 h (Figure 3D). Although PA + G25 also elevated VEGFA expression relative to G5, mean fluorescence intensity was lower than with PA alone, indicating that PA primarily drives VEGFA overexpression, while high glucose may partially modulate this effect (Figure 3C,D). Fluorescence frequency distribution analysis revealed dynamic shifts within the cell population. At 24 h, PA-treated cells exhibited a broad, platykurtic distribution (Figure 3E), reflecting high cellular heterogeneity with a wide range of expression levels extending into high-intensity values. The PA + G25 group showed a rightward shift relative to controls but with a narrower distribution than PA alone, lacking extremely high-intensity outliers (Figure 3E). By 48 h, the distribution profiles of both lipid-exposed groups (PA and PA + G25) became more uniform (leptokurtic) (Figure 3F), indicating that the surviving population is converging toward a high and stable VEGFA phenotype, despite a decrease in mean fluorescence intensity compared to 24 h, reflecting the loss of the most extreme hyperexpression outliers. Exposure to the osmotic control, mannitol (20 mM), showed no change in VEGFA expression (Figure S2).

To mathematically support the observed shifts in heterogeneity, a Gaussian Mixture Model (GMM) was employed as an ancillary analysis. This enabled the objective identification of subpopulations within the frequency distribution, revealing a distinct high-expressor phenotype in the PA and PA + G25 groups. Model selection based on the Bayesian Information Criterion (BIC) identified three optimal components (K = 3) for both G5 and G25 conditions at the analyzed time points (Table 1). Under these conditions, the population was primarily homogeneous; a dominant subpopulation (Sub-Pop 1) accounted for most cells and exhibited stable, low-intensity fluorescence, confirming that hyperglycemia alone does not alter the baseline phenotypic distribution of VEGFA expression. In contrast, PA exposure significantly increased phenotypic diversity, requiring 5 components (K = 5) to best fit the data at both 24 and 48 h (Table 1). At 24 h, PA treatment led to the appearance of two VEGFA hyper-expressing subpopulations (Sub-Pops 4 and 5), together accounting for about 40% of the total population (Table 1). By 48 h, although the 5-component structure persisted, the proportion of these extreme hyper-expressing cells declined, with a corresponding shift in the population toward intermediate/high intensities (Table 2). The combined treatment (PA + G25) showed a less complex population structure than PA alone, with optimal K values of 3 at 24 h and 4 at 48 h (Table 1 and Table 2). Importantly, the extremely high-intensity clusters seen under PA conditions were missing with high glucose present. Instead, the PA + G25 group displayed a shift toward intermediate fluorescence levels, indicating that high glucose partially reduces the extreme variability caused by PA.

**Figure 4:**
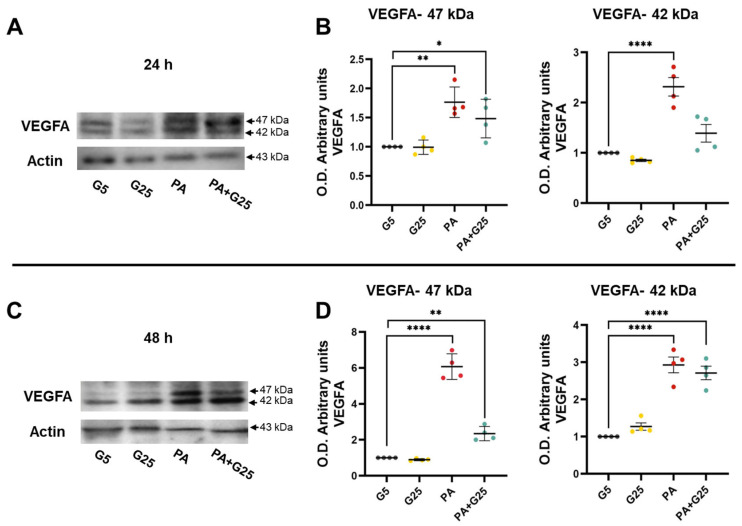
Palmitic acid increases VEGFA levels in MGCs. Relative expression levels were normalized to actin. (**A**,**C**) Representative Western blots. (**B**,**D**) Densitometric quantification of VEGFA (47 and 42 kDa). Values represent the mean ± SEM (*n* = 4 per group), performed in duplicate. Values are the mean ± SEM (*n* = 4 per group) carried out in duplicate. Normal glucose (G5), high glucose (G25), palmitic acid (PA), and the combination (PA + G25). Statistical significance relative to the G5 control is indicated as * *p* < 0.05, ** *p* < 0.01, and **** *p* < 0.0001. VEGFA (42 kDa) levels were significantly lower (*p* < 0.01) in the PA + G25 group compared to the PA group at 24 h. VEGFA (47 kDa) levels were significantly lower (*p* < 0.0001) in the PA + G25 group compared to the PA group at 48 h.

To confirm VEGFA expression through immunofluorescence, total VEGFA protein levels were measured by Western blot. Immunoblot analysis showed two bands at approximately 42 and 47 kDa across all experimental conditions (Figure 4A,C), likely representing dimeric and/or glycosylated VEGFA variants previously described in the literature [12,13]. Consistent with the immunofluorescence results, exposure to G25 did not change the expression of any VEGFA isoforms compared to G5 at the studied time points (Figure 4B). Conversely, treatment with PA caused a significant increase in both the 42 and 47 kDa bands at 24 and 48 h (Figure 4B,D). In cells treated with PA + G25, VEGFA upregulation was reduced compared to PA alone (Figure 4B,D), especially at 48 h for the 47 kDa isoform (Figure 4D). These findings support the conclusion that PA overload induces VEGFA overexpression.

### 2.3. Palmitic Acid Promotes Continuous VEGFA Release by MGCs

**Figure 5:**
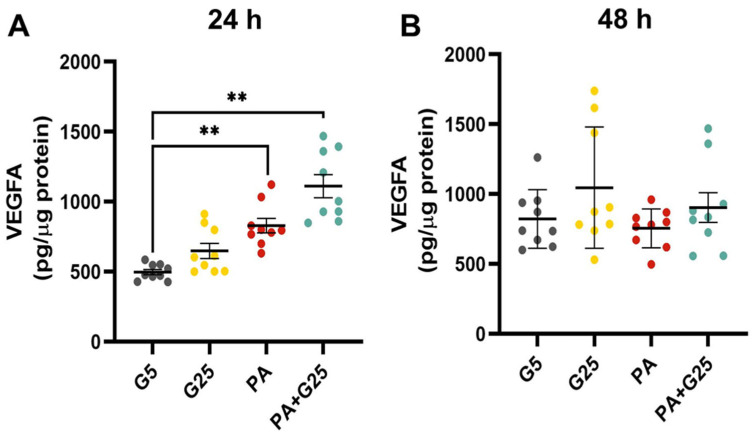
Palmitic acid promotes VEGFA secretion in MGCs: (**A**,**B**) VEGFA levels released into the culture medium were measured by ELISA at 24 and 48 h. Cells were exposed to control (G5), high glucose (G25), palmitic acid (PA), and the combination (PA + G25). Data are shown as the mean ± SEM from four independent experiments (*n* = 9). Statistical significance compared to the G5 control is indicated as ** *p* < 0.01. Additionally, a notable increase in VEGFA secretion was observed in the PA + G25 group compared to the PA group at 24 h (*p* < 0.01).

To assess whether intracellular VEGFA overexpression results in a functional paracrine response, we measured VEGFA release into the culture medium. Under G5 conditions, a baseline level of VEGFA release was observed, supporting its role in maintaining the retinal microenvironment. Exposure to G25 did not significantly change VEGFA release at the analyzed time points (Figure 5). In contrast, treatment with PA and PA + G25 triggered an acute release of VEGFA into the culture medium at 24 h (Figure 5B). However, by 48 h, no significant differences in VEGFA release were observed among the conditions. Since these values are normalized to total protein content to account for the reduction in cell density, these results suggest that while the secretion rate per unit of protein remains elevated in PA-treated groups, the statistical difference with control groups (G5 and G25) disappears, likely due to a progressive increase in their baseline VEGFA release over time.

### 2.4. Heterogeneous Overexpression of VEGFR2 in MGCs Exposed to Palmitic Acid

**Figure 6:**
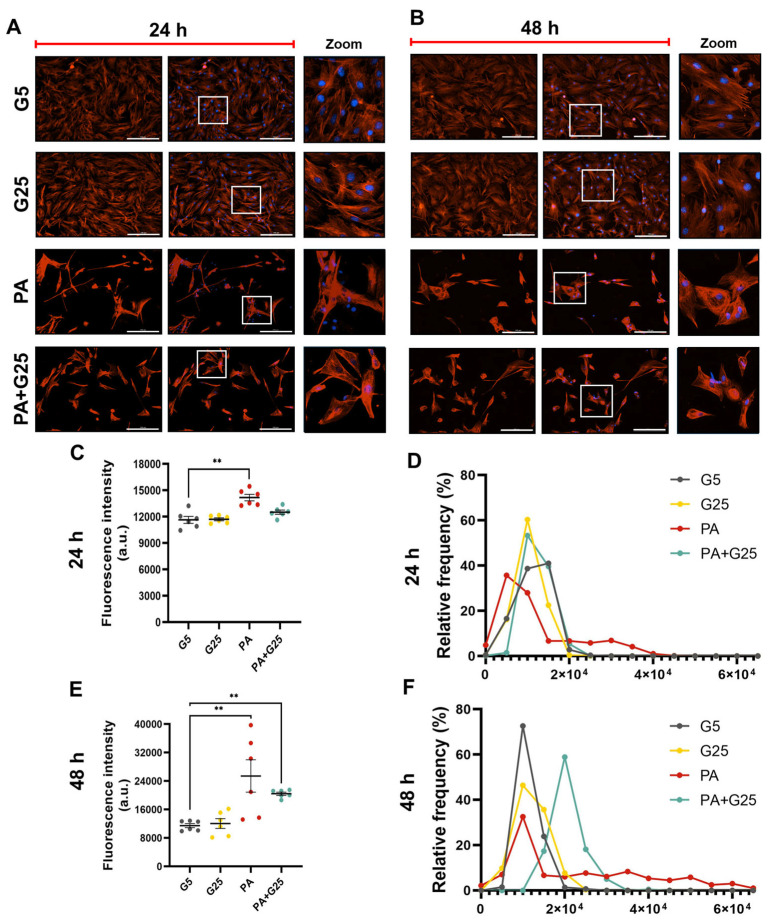
Palmitic acid causes variable VEGFR2 expression in MGCs: (**A**,**B**) Representative immunofluorescence images showing VEGFR2 (red) and nuclei (DAPI, blue) in cells exposed to the specified conditions for 24 and 48 h. (**C**–**E**) Quantification of average fluorescence intensity (arbitrary units, a.u.). Data are presented as mean ± SEM from six independent experiments (100 cells analyzed per experiment; *n* = 6). (**E**,**F**) Relative frequency distribution (%) of fluorescence intensity per cell (total pooled *n* = 600 cells). Cells were treated with normal glucose (G5), high glucose (G25), palmitic acid (PA), and the combination (PA + G25) at 24 and 48 h. Scale bars: 200 µm. Statistical significance compared to the G5 control is marked as ** *p* < 0.01. (**C**,**D**). Significant differences were observed between the PA and PA + G25 groups (*p* < 0.01) at 24 h.

After establishing the hypersecretory VEGFA phenotype, we assessed VEGFR2 expression, the primary receptor involved in angiogenic signaling, to determine whether MGCs respond to VEGFA in an autocrine fashion. Under G5 conditions, VEGFR2 exhibited positive labeling with a diffuse cytoplasmic distribution (Figure 6A,B). Consistent with the VEGFA results, hyperglycemia alone did not change VEGFR2 expression levels or its subcellular localization at the tested time points. In contrast, exposure to PA caused a significant increase in VEGFR2 from 24 to 48 h (Figure 6C,E). The PA + G25 treatment notably elevated its expression up to 48 h (Figure 6E). Analysis of the frequency distribution at 24 h showed that PA treatment induced phenotypic heterogeneity (platykurtic) (Figure 6D), with some cells overexpressing the receptor and others showing no labeling. The G5, G25, and AP + G25 conditions displayed more uniform (leptokurtic) distributions (Figure 6D). At 48 h, PA treatment increased heterogeneity, broadening the range of cells (platykurtic) and boosting receptor overexpression. The PA + G25 condition exhibited a uniform (leptokurtic) distribution with higher fluorescence intensity than G5 and G25 (Figure 6F). Overall, VEGFR2 expression in these cells indicates sensitivity to VEGFA-mediated autocrine signaling, which is heightened under PA exposure. Importantly, exposure to the osmotic control mannitol (20 mM) did not alter VEGFR2 expression, confirming that the effects observed were not due to osmotic stress (Figure S3).

To quantitatively evaluate the phenotypic heterogeneity observed in the immunofluorescence analysis, GMM clustering was applied to VEGFR2 fluorescence intensity datasets. At 24 h, the G5 condition showed three subpopulations, with average fluorescence intensities ranging from 7000 to 17,000 arbitrary units. The G25 exposure revealed four subpopulations, but the fluorescence intensities ranged from 6000 to 13,000 units, within the range observed in G5. PA treatment identified three subpopulations, one of which was characterized by extensive VEGFR2 overexpression. The PA + G25 condition was best described by two subpopulations with fluorescence ranges similar to those in G5. At 48 h, PA exposure significantly increased population complexity, producing six distinct subpopulations, four of which exhibited VEGFR2 overexpression, compared to G5. The PA + G25 treatment increased the number of subpopulations to three, with one overexpressing VEGFR2. Overall, the subpopulation analysis indicates dynamic, time-dependent changes in VEGFR2 expression in response to palmitic acid exposure, marked by the emergence of the receptor and suggesting increased VEGFR2 sensitivity.

### 2.5. Palmitic Acid Causes Early Sp1 Increase, While Combined Exposure with High Glucose Boosts Late HIF-1α Expression

**Figure 7:**
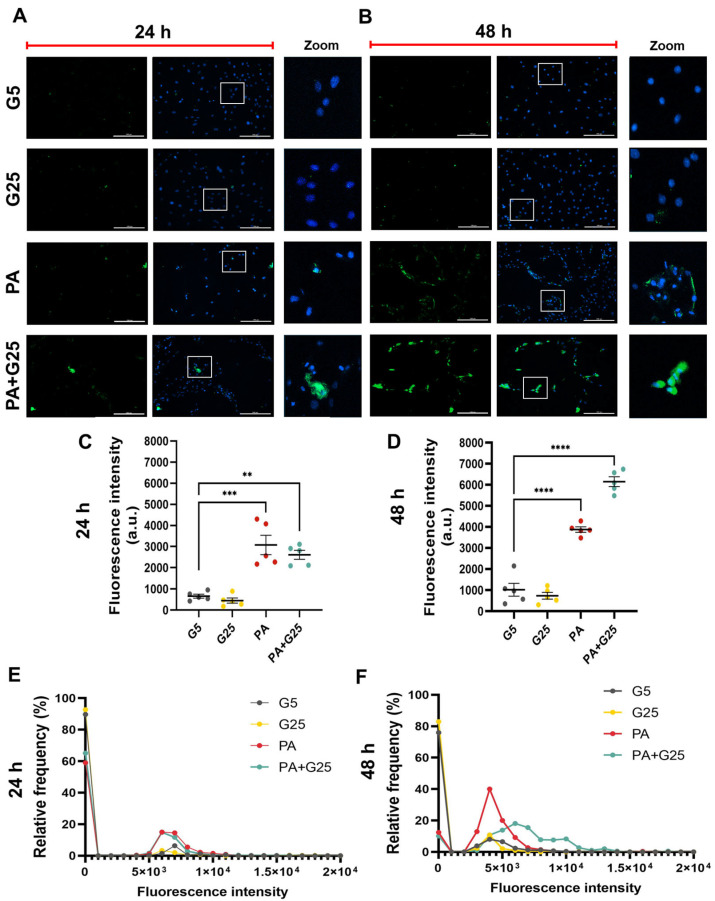
High glucose enhances palmitic acid-induced HIF-1α expression in MGCs during late stages: (**A**,**B**) Representative immunofluorescence images showing HIF-1α (green) and nuclei (DAPI, blue) in cells exposed to the indicated conditions for 24 and 48 h. (**C**,**D**) Quantification of mean fluorescence intensity (arbitrary units, a.u.). Data are presented as mean ± SEM from five independent experiments (100 cells analyzed per experiment; *n* = 5). (**E**,**F**) Relative frequency distribution (%) of fluorescence intensity per cell (total pooled *n* = 500 cells). Cells were treated with normal glucose (G5), high glucose (G25), palmitic acid (PA), and the combination (PA + G25) at 24 and 48 h. Scale bars: 200 µm. Statistical significance compared to the G5 control is indicated as ** *p* < 0.01, *** *p* < 0.001, and **** *p* < 0.0001. (**C**,**D**). Significant differences were observed between the PA and PA + G25 groups (*p* < 0.0001) at 48 h.

VEGFA overexpression can be triggered by increased transcriptional activity mediated by specific transcription factors. To explore this mechanism, we examined the expression of two factors involved in VEGFA transcription: hypoxia-inducible factor 1-alpha (HIF-1α) and Specificity protein 1 (Sp1). Under G5 and G25 conditions, HIF-1α showed no detectable immunoreactivity at either 24 or 48 h, indicating that hyperglycemia alone is not enough to stabilize HIF-1α under normoxic conditions (Figure 7A,B). In contrast, exposure to PA and PA + G25 caused an accumulation of HIF-1α. Quantitative analysis showed a significant increase in HIF-1α fluorescence intensity in PA-treated cells starting at 24 h (Figure 7C). Notably, the combined treatment (PA + G25) demonstrated a time-dependent synergistic effect. While HIF-1α levels were moderate at 24 h, they increased dramatically by 48 h, reaching levels significantly higher than those in PA alone (Figure 7D). This late-phase boost was supported by frequency distribution analysis, which displayed a strong rightward shift in the entire PA + G25 population toward higher fluorescence intensities, compared to the broader, more variable distribution seen in PA-treated cells (Figure 7E,F). 

**Figure 8:**
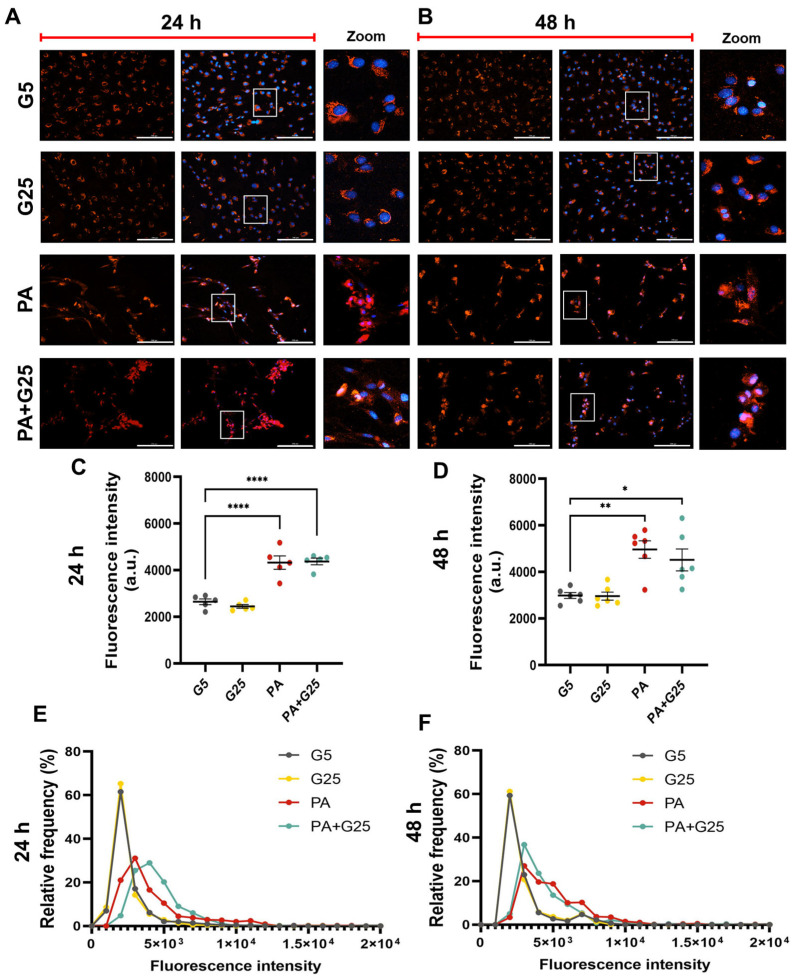
Palmitic acid increases SP1 expression in MGCs: (**A**,**B**) Representative immunofluorescence images of SP1 (red) and nuclei (DAPI, blue) in cells exposed to the indicated conditions for 24 and 48 h. (**C**,**D**) Quantification of mean fluorescence intensity (arbitrary units, a.u.). Data are presented as mean ± SEM from five independent experiments (100 cells analyzed per experiment; *n* = 5). (**E**,**F**) Relative frequency distribution (%) of fluorescence intensity per cell (total pooled *n* = 500 cells). Cells were exposed to normal glucose (G5), high glucose (G25), palmitic acid (PA), and the combination (PA + G25) at 24 and 48 h. Scale bars: 200 µm. Statistical significance compared to the G5 control is indicated as * *p* < 0.05, ** *p* < 0.01 and **** *p* < 0.0001.

HIF-1α did not show an increase after 24 h of exposure to PA and PA + G25, so we chose to evaluate SP1 as a potential early transcriptional regulator of VEGFA expression at early time points. Under G5, Sp1 exhibited basal expression with mainly perinuclear localization (Figure 8A,B). Treatment with G25 did not change SP1 expression at any of the analyzed time points. Exposure to PA and PA + G25 caused a significant increase in SP1 expression from 24 to 48 h, with similar frequency distributions in both conditions. These results demonstrate a biphasic transcriptional response that elevates VEGFA under PA, marked by early Sp1 activation at 24 h and increased Sp1 and HIF-1α levels at 48 h.

### 2.6. Palmitic Acid Activates Caspase-Dependent Apoptotic Pathways Involving Bax/Bcl-2 Dysregulation and Caspase-12 Cleavage

**Figure 9:**
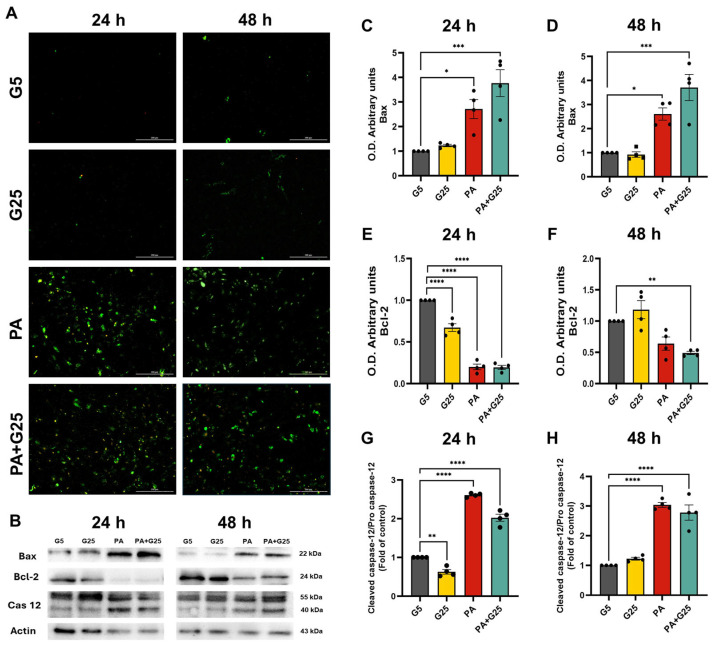
Palmitic acid causes apoptosis through joint mitochondrial and endoplasmic reticulum stress pathways: (**A**) Representative images showing executioner caspase 3/7 activity detected by a fluorogenic substrate (green) in cells exposed to the indicated conditions for 24 and 48 h. (**B**) Western blots of Bax, Bcl-2, Caspase-12 (Cas 12), and actin (loading control). Densitometric measurements of Bax (**C**,**D**) and Bcl-2 (**E**,**F**) expression levels. (**G**,**H**) Analysis of the Cleaved Caspase-12/Pro-Caspase-12 ratio as an indicator of ER stress. Expression levels were normalized to actin. Data are shown as mean ± SEM from four independent experiments (*n* = 4) performed in duplicate. Cells were treated with normal glucose (G5), high glucose (G25), palmitic acid (PA), and the combination (PA + G25) at 24 and 48 h. Scale bars: 200 µm. Statistical significance compared to the G5 control is marked as * *p* < 0.05, ** *p* < 0.01, *** *p* < 0.001, and **** *p* < 0.0001. Significant differences were observed in Cleaved Caspase-12/Pro-Caspase-12 ratio between the PA and PA + G25 groups (*p* < 0.0001) at 24 h.

Finally, to clarify the mechanism behind the significant decrease in cell viability described in Section 2.1, we investigated whether MGCs undergo apoptosis. First, we measured the activation of executioner caspases 3 and 7 using a fluorogenic substrate-based assay. As expected, cells exposed to G5 and G25 conditions showed minimal caspase activity at 24 and 48 h (Figure 9A). In contrast, PA and PA + G25 treatments induced strong activation of effector caspases (Figure 9A).

To identify the upstream pathways initiating this apoptotic cascade, we next examined the expression of Bcl-2 family proteins that regulate mitochondrial integrity. Western blot analysis revealed a clear imbalance between pro- and anti-apoptotic factors. Lipotoxic conditions (PA and PA + G25) caused a significant increase in the pro-apoptotic protein Bax (Figure 9C,D) and a marked decrease in the anti-apoptotic protein Bcl-2 (Figure 9E,F) at both 24 and 48 h compared to controls. This shift strongly suggests activation of the intrinsic mitochondrial apoptotic pathway. Under G25 conditions, Bcl-2 levels decreased at 24 h and were restored at 48 h (Figure 9E,F). Furthermore, since fatty acid overload disrupts endoplasmic reticulum (ER) homeostasis, we evaluated caspase-12 processing, a specific marker of ER stress-induced apoptosis. Densitometric analysis showed that PA and PA + G25 treatments significantly increased the cleaved Caspase-12/pro-Caspase-12 ratio compared to the G5 (Figure 9G,H). In cells exposed to G25, Caspase-12 levels were significantly reduced at 24 h (Figure 9G) and returned to G25 levels at 48 h (Figure 9G). This reduction, along with the decrease in Bcl-2 expression, suggests that G25 induces a state of susceptibility. Collectively, these results indicate that the cytotoxicity described in Section 2.1 is apoptotic rather than necrotic, driven by a dual mechanism involving mitochondrial dysregulation (Bax/Bcl-2 imbalance) and ER stress (Caspase-12 activation), specifically triggered by lipotoxicity (Figure S4).

## 3. Discussion

Diabetes mellitus is a metabolic disorder mainly characterized by persistent high blood sugar. While hyperglycemia is the primary feature of diabetes, other factors also contribute to the development of diabetes-related complications [1]. Type 2 diabetes (T2D) is strongly linked to being overweight and obese; in addition to high blood sugar, patients with T2D often experience dyslipidemia, which includes low HDL levels and increased triglycerides, cholesterol, and free fatty acids [10]. Research on diabetes complications has frequently focused on blood sugar levels without acknowledging the significant role of other metabolic stressors, such as elevated triglycerides and free fatty acids (FFA).

Diabetic retinopathy (DR) is one of the most common microvascular complications of diabetes. Clinically, retinopathy is marked by lesions in the retinal blood vessels. In the early stages, microaneurysms, hemorrhages, and hard exudates appear. Later stages involve neovascularization and macular edema [11,12]. Although intensive glycemic control and long-term HbA1c reduction have been demonstrated in numerous clinical trials as key strategies for stopping DR progression, this approach has limitations [13,14,15,16]. In patients with longstanding diabetes, strict glycemic control often cannot reverse existing damage and may even worsen eye disease or raise mortality risk [17,18]. Managing dyslipidemia has become an effective treatment; some reports show that fenofibrate provides strong retinoprotective effects, whereas statins have shown inconsistent or minimal efficacy in preventing DR [19,20,21,22]. Therefore, it is crucial to analyze the specific role of lipids and their interaction with hyperglycemia in the pathological mechanisms underlying DR. While the effects of high glucose levels on Müller glial cell (MGC) physiology have been extensively studied in vitro, little is known about how fatty acids interact with hyperglycemia, which remains poorly understood.

Our findings reinforce the hypothesis that dyslipidemia plays a role as critical as, or even more significant than, hyperglycemia in the progression of diabetic retinopathy. While clinical management traditionally focuses on glycemic control, the potent activation of proangiogenic signaling by PA suggests that lipid stress is a key determinant of cellular dysfunction. This implies that therapeutic strategies that do not address the lipid component may be insufficient to halt angiogenic change in the diabetic retina.

In this study, we examined the cytotoxic effects of palmitic acid (PA), hyperglycemia (G25), and their combination (PA + G25). Exposure to G25 alone did not cause significant cytotoxicity at the time points assessed (Figure 1 and Figure 2). This adds to the ongoing debate in the literature regarding glucose-induced cell death in MGC [23,24,25,26,27,28,29,30]; differences among studies likely result from variations in experimental conditions, such as cell source (primary cultures versus immortalized lines), glucose concentration, and exposure duration. While lipotoxicity has been shown in various cell types [31,32,33], this study is among the first to specifically investigate the susceptibility of MGCs to saturated fatty acid overload. Exposure to PA and PA + G25 significantly reduced cell viability starting at 24 h (Figure 2). Since G25 exposure did not exhibit cytotoxicity, this indicates that the toxicity from PA + G25 is primarily due to lipid stress rather than hyperglycemia.

Our data showed activation of apoptosis-related pathways in MGCs exposed to PA. At 24 h, both PA and PA + G25 treatments triggered apoptosis markers, including increased activity of caspases 3 and 7, an increase in BAX (pro-apoptotic), and a decrease in Bcl-2 (anti-apoptotic) (Figure 9). The specific cleavage of Caspase-12, a hallmark of endoplasmic reticulum (ER) stress-induced apoptosis, was significantly elevated in PA-exposed cells but absent in those treated with high-glucose alone (Figure 9). The simultaneous reduction in Bcl-2 and Caspase-12 in the G25 group at 24 h could suggest a primed state of susceptibility in MGCs. This early weakening of the anti-apoptotic defense, in the absence of immediate caspase activation, indicates a transitional period where the apoptotic cascade is not yet fully engaged. However, this state likely increases long-term glucotoxic susceptibility, thereby facilitating cell death under sustained, combined, or synergistic stress, as observed in the PA + G25 treatment. Lipid metabolism mainly occurs in the mitochondria through β-oxidation and in the ER, where fatty acids, phospholipids, ceramides, and lipid droplets are produced [34,35]. The simultaneous activation of apoptotic pathways involving these two organelles suggests that PA affects the metabolic functions of both mitochondria and the ER, ultimately leading to cell death. Detailed characterization of the cell death pathway is fundamental to understanding the pathogenesis of diabetic retinopathy. Distinguishing between general apoptotic markers and those specifically linked to ER stress, such as caspase-12, is highly relevant for the development of targeted neuroprotective therapies. Our findings suggest that while hyperglycemia alone initially weakens the anti-apoptotic threshold, the addition of palmitic acid is necessary to trigger the ER stress-mediated apoptotic cascade. Identifying this process provides a framework for pharmacological intervention.

Vascular endothelial growth factor A (VEGFA) is a crucial angiogenic factor involved in the development of DR [36,37,38]. In the retina, MGCs are a major source of VEGFA in both rodent models and people with diabetes [39,40,41,42,43]. To evaluate the angiogenic potential of MGCs under metabolic stress, we examined VEGFA expression and secretion. Under normal glucose conditions, MGCs showed baseline VEGFA levels and release (Figure 3, Figure 4 and Figure 5); this baseline release might be essential for maintaining retinal homeostasis and the integrity of the iRBR. Exposure to G25 did not cause significant changes in VEGFA expression or secretion compared to G5. In contrast, treatments with PA and PA + G25 significantly increased VEGFA expression and release from 24 to 48 h. However, phenotypic analysis of VEGFA fluorescence intensity per cell revealed distinct subpopulation patterns at the examined time points (Figure 3, Figure 4 and Figure 5).

Subpopulation analysis using the Gaussian Mixture Model (GMM) serves as an exploratory quantitative bridge between qualitative observations of frequency dispersion and the biological reality of MGC heterogeneity. While based on fluorescence intensity, the convergence of these groups in independent replicates suggests a reproducible phenotypic shift toward angiogenic signaling. GMM analysis at 24 h of exposure identified three subpopulations under G5 and G25 conditions, with approximately 80% of the population residing in a low-fluorescence group (≈3500) exhibiting a leptokurtic distribution. Palmitic acid promoted the emergence of five subpopulations, including three with overexpression (>8000) and one without a label, resulting in a platykurtic distribution characterized by high variability. The combined treatment (PA + G25) revealed three subpopulations, with the largest group showing medium fluorescence intensity (≈5000) and a mesokurtic distribution (Table 1). The highly expressing subpopulations in the PA treatment may suggest a reactive phenotype capable of withstanding lipotoxicity and activating focal angiogenic signaling. Conversely, the unlabeled subpopulation might correspond to cells that are especially vulnerable to the cytotoxic effects of PA. 

At 48 h, the GMM analysis identified three subpopulations under G5 and G25 conditions, with average fluorescence intensities similar to those at 24 h, and maintained a leptokurtic distribution. PA exposure also revealed five subpopulations, with the dominant one concentrated at high fluorescence intensities (>5000), while the population lacking fluorescence decreased, resulting in a mesokurtic fluorescence distribution. PA + G25 treatment increased the number of subpopulations to four, including one with undetectable fluorescence intensity. The most prominent subpopulation showed a leptokurtic distribution at high fluorescence intensities (Table 2). Overall, subpopulation analysis revealed significant variability in VEGFA expression, which may be key to understanding the pathogenesis of DR. In the retinal context, these results suggest that PA overload promotes MGC death, while the surviving cell population exhibits high VEGFA expression. This scenario could lead to focal regions of glial hyperactivity that precede the development of a chronic, widespread pro-angiogenic state driven by the surviving cells.

The VEGFA released by MGCs likely acts in a paracrine manner on retinal endothelial cells, promoting angiogenesis during DR progression. The dynamic changes in VEGFA subpopulations over time may reflect the selection of a secretory phenotype reinforced by autocrine VEGFA signaling within MGCs. Under basal conditions, VEGF receptor 2 (VEGFR2) was expressed in MGCs, with distribution throughout the cell body. Exposure to high glucose concentrations did not alter overall fluorescence intensity but did change the number of subpopulations (Figure 6). Four subpopulations appeared at 24 h and two at 48 h (Table 3 and Table 4). Unlike VEGFA expression, which showed no change in expression or subpopulation numbers under high glucose, the increase in receptor subpopulations suggests altered cell sensitivity to VEGFA. PA increased VEGFR2 fluorescence intensity (Figure 6), but the number of subpopulations varied over time. At 24 h, three subpopulations were observed, including one with high fluorescence intensity. By 48 h, the number of subpopulations increased to six, with three exhibiting high fluorescence intensity (Table 3 and Table 4). 

These overexpression profiles may indicate a positive feedback loop where increased VEGFA levels enhance the selection of cells with higher receptor expression, thus fostering a localized angiogenic niche. Notably, the combined treatment exhibited a complex temporal pattern in receptor expression. Our subpopulation analysis showed that while PA alone caused significant and heterogeneous VEGFR2 overexpression, with high-intensity outliers appearing as early as 24 h, elevated glucose levels (PA + G25) initially suppressed this response (Figure 6). The early decrease in PA-induced VEGFR2 overexpression by high glucose at 24 h suggests a metabolic rerouting of excess saturated fatty acids. This buffering likely results from increased availability of glycolytic intermediates, such as glycerol-3-phosphate, which promote the esterification of lipotoxic PA into neutral triglycerides stored in lipid droplets. By sequestering PA away from mitochondria and endoplasmic reticulum, MGCs temporarily avoid activating apoptotic signals and extreme phenotypic changes. However, the convergence toward a high-expression phenotype at 48 h indicates that this protective mechanism is limited and eventually overwhelmed by chronic glucolipotoxicity and persistent pro-angiogenic signaling.

Although our experimental design did not directly assess tube formation or endothelial migration, the simultaneous upregulation of VEGFA secretion and its receptor in MGCs indicates a significant enhancement of pro-angiogenic signaling. In the retina, MGCs span all layers and are in direct contact with the vasculature; therefore, the autocrine and paracrine loop we observed (VEGFA/VEGFR2) could serve as a continuous stimulus for pathological neovascularization. Future studies using co-cultures with retinal endothelial cells will be essential to functionally validate how this MGC-derived secretome triggers the physical assembly of new vessels, a process inferred here from the robust expression of these canonical angiogenic factors.

Transcription factors regulate VEGFA levels. Hypoxia-inducible factor 1 subunit alpha (HIF-α) and specific protein 1 (SP1) are recognized as key regulators in angiogenesis, promoting VEGFA overexpression [44]. HIF-α is a transcription factor that activates in response to hypoxia. Under normoxic conditions, the proline residues of HIF-α are hydroxylated by the prolyl-hydroxylase complex, creating ubiquitination target sites that lead to proteasomal degradation. During hypoxia, proline hydroxylation is inhibited, preventing HIF-1α degradation. The stabilization and accumulation of HIF-1α enable its translocation to the nucleus, where it dimerizes with HIF-1 and activates the transcription of genes involved in angiogenesis and metabolic adaptation [45]. Besides hypoxia, HIF-1 levels can also be regulated by metabolic signaling pathways such as PI3K/Akt, MAPK/ERK, and the buildup of metabolic intermediates like succinate, which inhibit prolyl-hydroxylases (PHDs), thus regulating HIF levels [46]. In this study, we observed that exposure to high glucose concentrations did not increase HIF-α levels, consistent with the unchanged VEGFA levels under these conditions. Exposure to PA alone and in combination increased HIF-α levels, with responses varying at different time points. At 24 h, only a small percentage of cells showed increased HIF-α, while most showed no detection of HIF-α (Figure 7).

In contrast, at 48 h, most of the population showed detection of HIF-α. These results can be seen as a biphasic response: at 24 h, MGCs may mainly activate acute stress pathways in response to PA without fully triggering an alternative mechanism that stabilizes HIF-α. However, by 48 h, when the surviving cells have developed a phenotype with overexpression of both VEGFA and its receptor, HIF-α activation occurs—possibly to sustain the positive feedback loop of the VEGFA/VEGFR2 axis. Notably, the combined treatment (PA + G25) caused a greater increase in HIF-α levels at 48 h than PA alone. This pattern differs from VEGFA and VEGFR2 expression, where PA + G25 increased levels comparable to or lower than those under PA alone. This suggests that specific adaptations to different metabolic stressors may, over longer periods, be crucial in selecting cells with an angiogenic or inflammatory phenotype.

Sp1 is a transcription factor that controls cellular functions like proliferation, differentiation, and angiogenesis, and is broadly associated with tumor development [47]. Several studies have shown that Sp1 influences a part of basal VEGF expression [48,49,50,51]. In this study, we found that basal Sp1 expression occurs in cells under normal glucose conditions. Consistent with previous findings, high glucose levels did not change Sp1 expression or its location within the cell. Exposure to PA and PA + G25 increased Sp1 levels from 24 to 48 h (Figure 8). Sp1 activity is regulated by various post-translational modifications, including phosphorylation, acetylation, and glycosylation, which can be triggered by metabolic stress caused by PA and high glucose [47]. Overall, our data suggest a biphasic transcriptional regulation involving HIF1-α and Sp1, promoting an angiogenic phenotype in MGCs. We observed that Sp1 responds early to palmitic acid stress, enhancing VEGFA expression at 24 h. Later, during prolonged exposure, stabilization of HIF1-α strengthens the VEGFA/VEGFR2 feedback loop, maintaining pro-angiogenic signaling.

The results of this study indicate the presence of phenotypes that enhance VEGFA/VEGFR2 signaling in response to PA-induced metabolic stress. Penn’s research group previously reported similar findings, demonstrating that exposing primary human MGC cultures to palmitic acid, along with high glucose, increased the expression of genes related to intracellular lipid signaling, inflammation, and angiogenesis [52]. However, exposure to high glucose alone did not cause significant changes in genes associated with these pathways. These observations emphasize the importance of analyzing lipid changes in the retina as key factors in DR progression. 

It is important to note that current findings on the effects of hyperglycemia should be interpreted with caution. Our study focused on 24 and 48 h exposure periods, representing a short-term model of metabolic stress. Therefore, the absence of significant changes in angiogenic signaling under hyperglycemic conditions during this period does not imply that hyperglycemia has a limited role in the overall pathogenesis of diabetic retinopathy. Since glucose-induced damage is typically time-dependent and cumulative, further studies with longer exposure periods would be needed to fully characterize the long-term contribution of hyperglycemia to MGCs dysfunction in this model.

Despite the observed findings, the use of 250 μM PA represents a model of acute lipotoxicity rather than chronic low-grade metabolic stress. While this concentration is used in the literature to induce measurable inflammatory/angiogenic changes in short-term cultures [52,53,54], it does not replicate the chronic exposure that occurs in diabetic retinopathy. Therefore, future studies could focus on evaluating lower concentrations over extended periods to model chronic metabolic adaptation, a necessary step to translate these findings into a clinical context.

Although this work addresses the impact of palmitic acid on the selection of a pro-angiogenic phcapturn MGCs, our study, over a 48 h period, encompasses an acute and transient stress phase; chronic in vivo exposure likely involves complex compensatory mechanisms. Furthermore, while immunofluorescence provides invaluable spatial data on protein localization and expression levels, it remains a semi-quantitative approach that should be complemented by transcriptomic analyses such as single-cell RNA sequencing (scRNA-seq) to molecularly profile these VEGFA/VEGFR2 super-expressing subpopulations. A full understanding of the observed phenotypes would require, for example, assessing the functional impact of conditioned media on MGCs under different metabolic stress conditions and evaluating their angiogenic potential in endothelial cells. Extrapolating these results to in vivo models will be crucial to determining whether lipid-lowering therapies or specific ER stress inhibitors can prevent the increase in VEGFA/VEGFR2, potentially offering a new pharmacological approach for patients who do not respond to conventional anti-VEGF treatments.

## 4. Materials and Methods

### 4.1. Primary Müller Cell Culture

Retinal MGCs were isolated from postnatal (4–7 days) *CD1* mice. Enucleated eyes were placed in Dulbecco’s Modified Eagle Medium F12 (DMEM F12; Sigma-Aldrich, Burlington, MA, USA, #51445C) supplemented with 1% penicillin/streptomycin (15140122, Gibco, Grand Island, NY, USA) and 10% fetal bovine serum (S1620, Biowest, Brandenton, FL, USA) (DMEM F12-FBS). Eyes were protected from light and incubated with gentle rotation overnight at room temperature. Next, the eyes were treated with 0.25% trypsin-EDTA (Gibco, Grand Island, NY, USA) for 5 min; trypsin was then inactivated with DMEM F12-FBS. The retinas were carefully dissected and mechanically dissociated in DMEM F12-FBS before being seeded in 60 mm Petri dishes. Cells were cultured in DMEM F12-FBS supplemented with 800 ng/mL human epidermal growth factor (hEGF) at 37 °C in a humidified atmosphere of 95% air and 5% CO_2_. After three days in culture, the monolayer was rinsed to remove any attached non-glial cells. For passaging, the cells were incubated with 0.25% trypsin at 37 °C for five minutes. Trypsin was then inactivated with DMEM F12-FBS. The cells were mechanically resuspended, centrifuged at 500× *g* for 5 min at room temperature, and the pellet was resuspended in 1 mL of DMEM F12-FBS. Cell counts were performed using a Neubauer chamber, and the cells were seeded onto culture plates as required for the experiment. The cells used for all experimental conditions were from the third passage.

### 4.2. Experimental Conditions

MGCs were exposed to DMEM F12-FBS with normal (5 mM; G5) or high glucose (25 mM; G25), palmitic acid (250 μM; PA), and palmitic acid with high glucose (PA + G25) for 24 and 48 h. The culture medium was not changed during the treatment periods. The osmotic control medium consisted of 5 mM glucose supplemented with 20 mM mannitol. 

### 4.3. CCK8 Assay

MGCs were cultured in 96-well plates with 4000 cells per well and subjected to various experimental conditions (100 μL/well). After each exposure period, 10 μL of CCK-8 reagent (MCE, HY-K0301, Monmouth, NJ, USA) was added to each well and incubated for 4 h at 37 °C. The absorbance at 450 nm was measured using a multiplate reader (BioTek, Winooski, VT, USA, Epoch 2 microplate spectrophotometer). Cell viability was expressed as the percentage of the optical density relative to cells exposed to G5.

### 4.4. Trypan Blue Stain Assay

MGCs were cultured on 12-well plates with 16,000 cells per well. The cells were exposed to various experimental conditions and then detached using 0.25% trypsin for 5 min. Trypsin was neutralized with DMEM F12-FBS in a 1:1 ratio. The suspended cells were incubated with 0.4% trypan blue solution (Sigma-Aldrich, Burlington, MA, USA) at a 1:1 ratio. Cell counts were performed using a Neubauer chamber, and cell viability was assessed through trypan blue exclusion. Cell viability was calculated as the percentage of living cells for each condition (% living cells = (number of living cells/total cells) × 100).

### 4.5. Immunofluorescence

Cells were cultured on 48-well plates with 8000 cells per well and fixed using 4% paraformaldehyde (*w*/*v* in PBS) for 20 min at room temperature. They were rinsed three times for 10 min each with PBS. Samples were permeabilized with 0.3% Triton-X100 in PBS for 30 min. Then, the cells were blocked with 1% BSA in PBS for 1 h at room temperature. They were incubated overnight at 4 °C with the following primary antibodies: VEGFA (1:300, Mouse, Santa Cruz, Paso Robles, CA, USA, SC-7269); VEGFR2 (1:300, Mouse, Santa Cruz, SC-393163); SP1 (1:300, Mouse, Santa Cruz, SC17824); HIF-1α (1:300, Rabbit, Cell Signaling, Danvers, MA, USA, 361695). Afterward, cells were washed three times with PBS and incubated for two hours at room temperature protected from light with secondary antibodies: Alexa Fluor 647-conjugated goat anti-mouse (1:500; A21235, Thermo Scientific, Waltham, MA, USA), Alexa Fluor 488-conjugated goat anti-mouse (1:500; Abcam, Waltham, MA, USA), and Alexa Fluor 488-conjugated goat anti-rabbit (1:500; Thermo Fisher). Cell nuclei were stained with 300 nM DAPI (D4592, Sigma) for 10 min at room temperature. The samples were washed three times for 10 min each with PBS and stored at 4 °C in PBS until further imaging. Images were captured using a BioTek Cytation 5 Cell Imaging Multimode Reader (BioTek Instruments, Winooski, VT, USA), and fluorescence was quantified with the Gen5™ software version 3.15 (BioTek Instruments, Winooski, VT, USA).

### 4.6. Caspase-3/7 Activity Assay

MGCs were seeded in 48-well plates and subjected to the described experimental conditions. The activation of executioner caspases was detected using the CellEvent™ Caspase-3/7 Green Detection Reagent (Cat. No. C10740; Thermo Fisher Scientific, Waltham, MA, USA). After treatment, cells were incubated with the reagent at a final concentration of 5 µM for 30 min at 37 °C, protected from light. Then, cells were gently washed twice with PBS. Samples were immediately visualized and imaged using a BioTek Cytation 5 Cell Imaging Multimode Reader. Image acquisition was performed with Gen5™ software.

### 4.7. Live/Dead Cell Viability Assay

For the Live/Dead assay, the LIVE/DEAD™ Cell Imaging Kit (488/570) (Thermo Fisher Scientific) was used according to the manufacturer’s instructions. MGC cultures were incubated with a staining solution containing both live (Calcein AM) and dead (Ethidium homodimer) cell markers for 3 h. The cells were kept in PBS during image acquisition.

### 4.8. Western Blot

MGCs were seeded to confluency in 60 mm Petri dishes (Corning, Union City, CA, USA). After treatments, cells were rinsed with cold PBS and scraped to dislodge them. Cell samples were homogenized in RIPA lysis buffer (50 mM Tris-HCl, pH 8.0, 150 mM NaCl, 1% NP-40, 0.5% sodium deoxycholate, 1 mM EDTA, 2% Triton X-100, 0.2% SDS, and protease–phosphatase inhibitors). The samples were frozen at −80 °C overnight, then homogenized again and centrifuged at 17,000× *g* for 30 min at 4 °C. Supernatants were collected, and protein concentrations were measured using the BCA assay. Equal amounts of total protein (25 µg per sample) were mixed with Laemmli’s sample buffer, boiled for 5 min, and separated by 10% SDS-PAGE alongside molecular weight markers (PageRuler™ Plus; 00656499 (Thermo Fisher Scientific, Waltham, MA, USA)). Proteins were transferred onto polyvinyl difluoride membranes (Millipore Corp, Jaffrey, NH, USA) following standard protocols. The membranes were stained with Ponceau S to verify equal protein loading across all lanes. Nonspecific protein binding sites were blocked with 5% nonfat milk in TBS-Tween (0.1% Tween 20, 20 mM Tris–HCl, 136 mM NaCl, pH 7.6) for 2 h at room temperature. Subsequently, membranes were incubated overnight at 4 °C with primary antibodies diluted in 0.25% BSA in TBS-T: VEGFA (1:300, Santa Cruz, SC-7269); VEGFR2 (1:300, Santa Cruz, SC-393163); Bax (1:500, Santa Cruz, SC17824); Bcl2 (1:500, Santa Cruz, SC17824); Caspase 12 (1:500, Santa Cruz, SC17824); Actin (1:1000, Cell Signaling, 361695). The next day, membranes were incubated for 3 h at room temperature with an HRP-conjugated secondary antibody (1:8000; Immobilon Western Chemiluminescent HRP Substrate, Millipore). Signal detection was performed using chemiluminescence (Western Bright ECL HRP substrate, Advansta, San Jose, CA, USA). Immunoblots were scanned and quantified with Molecular Image ChemiDoc™ XRS+ using Image Lab™ software version 6.1. Protein levels were normalized to their respective loading control (actin). The relative protein levels under experimental conditions were calculated as the optical density of the samples relative to the control (G5) on the same Western blot.

### 4.9. ELISA

MGCs were cultured in 48-well plates and subjected to experimental treatments or controls for 24 and 48 h. The cell culture media were centrifuged and stored at −20 °C until processing. On the day of the experiment, the media were thawed, and the manufacturer’s protocol for the mouse VEGF-A ELISA (#RAB509, Sigma-Aldrich, Burlington, MA, USA) was followed. Absorbance was measured using a microplate reader (BioTek, Epoch 2 microplate spectrophotometer, Winooski, VT, USA), and VEGF-A concentrations were determined by interpolation from the standard curve, with an r2 ≥ 0.99.

### 4.10. Gaussian Mixture Models Clustering

The variability in cellular expression of VEGFA and VEGFR2 was analyzed using Gaussian Mixture Models (GMM). Unlike traditional descriptive statistics, GMM allows for a probabilistic separation of the fluorescence data into K distinct subpopulations. Data processing and analysis were conducted using Python (v3.10.16) and the scikit-learn library (v1.3.0). Raw fluorescence data from all biological replicates (*n* = 6) were combined and reformatted into a long structure, excluding null values. To ensure equal influence of variance and reduce numerical instability during Expectation-Maximization (EM), data for each experimental condition were normalized with Z-score standardization before model fitting, according to the equation:

$$Ζ=\;\frac{\chi -\mu }{\sigma }$$

where χ is the raw intensity, μ is the sample mean, and σ is the sample standard deviation. For model selection, we iteratively fitted GMMs with component counts ranging from 1 to 6 for each condition. We assumed unconstrained covariance matrices (full covariance) to allow for flexible cluster shapes. The optimal number of subpopulations (K) was determined by minimizing the Bayesian Information Criterion (BIC), which penalizes model complexity to prevent overfitting.

To ensure global convergence and reproducibility, the EM algorithm was initialized with 10 random starts, using a convergence tolerance of 10^−3^ and a fixed random seed. After fitting the model, parameters (means and covariances) were inverse-transformed to the original fluorescence scale for biological interpretation. Finally, the proportions of cells in each subpopulation were measured and compared across groups.

### 4.11. Data Presentation and Statistics

All data are expressed as the mean ± SEM and analyzed using GraphPad Prism 8 (GraphPad Software, La Jolla, CA, USA). The normal distribution of data was checked with the Shapiro–Wilk test. Levene’s test was used to evaluate the homogeneity of variance among groups. Statistical significance was determined with a one-way ANOVA followed by Tukey post hoc tests for multiple comparisons. For Western blot analysis, data were expressed as fold-change relative to the control group. A *p*-value ≤ 0.05 was considered statistically significant between the control and experimental groups. Statistical significance compared to the control (fixed at 1.0) was assessed using a one-sample *t*-test. Specific details for each experiment are provided in the corresponding figure legends.

## 5. Conclusions

This work shows that hyperlipidemia (palmitic acid) is a key trigger of an angiogenic secretory phenotype in MGCs. Using probabilistic subpopulation analysis, we identified a dynamic phenotypic profile marked by early heterogeneity, with potential clusters of hyperreactive cells at 24 h, followed by a stabilization into a persistent proangiogenic signaling at 48 h. These results indicate that the progression of diabetic retinopathy is driven by the selection of specific cellular phenotypes, which maintain harmful signaling through autocrine mechanisms in response to metabolic stress.

## Figures and Tables

**Table 1:** Quantitative parameters and phenotypic distribution of VEGFA-expressing subpopulations identified by Gaussian Mixture Model (GMM) analysis at 24 h.

Condition	Optimal K	Sub-Pop ID	Proportion (%)	Mean Int. Flu (μ)	Sta. Dev (σ)
G5	3	1	83.3	3372.32	351.26
		2	15.0	4675.73	636.55
		3	1.6	5961.50	2927.11
G25	3	1	80.7	3589.84	389.72
		2	18.7	4892.39	796.98
		3	0.5	9811.33	377.05
PA	5	1	6.9	0	4.53
		2	13.6	5812.09	563.39
		3	38.9	8415.02	964.59
		4	26.4	12,237.98	1587.1
		5	13.9	16,746.97	3353.17
PA + G25	3	1	63.3	4689.53	358.05
		2	32.6	5887.9	558.91
		3	4.0	8370.7	1479.77

Data represent the probabilistic deconvolution of cellular fluorescence intensity into discrete subpopulations. The optimal number of components (K) for each experimental condition was determined by minimizing the BIC. Conditions include normal glucose (G5), high glucose (G25), palmitic acid (PA), and the combination (PA + G25). Parameters include the mixing proportion (%), the inverse-transformed mean fluorescence intensity (μ), and the standard deviation (σ). *n* = 600 cells.

**Table 2:** Quantitative parameters and phenotypic distribution of VEGFA-expressing subpopulations identified by GMM analysis at 48 h.

Condition	Optimal K	Sub-Pop ID	Proportion (%)	Mean Int. Flu (μ)	Sta. Dev (σ)
G5	3	1	64.0	3911.14	277.64
		2	27.0	4639.85	421.84
		3	8.8	5879.19	1312.17
G25	3	1	64.1	4161.9	256.29
		2	29.0	4845.11	355.46
		3	6.7	5545.2	1868.75
PA	5	1	0.5	0	2.25
		2	56.9	5956.29	853.32
		3	29.1	7909.67	872.24
		4	11.0	10,454.87	1287.49
		5	2.3	15,585.53	2622.59
PA + G25	4	1	0.3	0	1.28
		2	53.7	4907.8	436.58
		3	33.6	6218.66	655.30
		4	12.2	7969.04	1376.27

Data represent the probabilistic deconvolution of cellular fluorescence intensity into distinct subpopulations. The optimal number of components (K) for each experimental condition was determined by minimizing the BIC. Parameters include the mixing proportion (%), the inverse-transformed mean fluorescence intensity (μ), and the standard deviation (σ). Normal glucose (G5), high glucose (G25), palmitic acid (PA), and the combination (PA + G25). *n* = 600 cells.

**Table 3:** Quantitative parameters and phenotypic distribution of VEGFAR2-expressing subpopulations identified by Gaussian Mixture Model (GMM) analysis at 24 h.

Condition	Optimal K	Sub-Pop ID	Proportion (%)	Mean Int. Flu (μ)	Sta. Dev
G5	3	1	30.8	7417.83	1145.02
		2	61.4	12,902.78	1265.97
		3	7.7	16,947.87	3216.17
G25	4	1	12.2	5929.29	440.86
		2	19.2	8298.52	794.64
		3	50.9	11,755.42	1110.26
		4	17.5	12,879.90	2475.44
PA	3	1	60.3	7323.81	935.29
		2	23.3	16,403.54	5558.90
		3	16.2	30,625.02	4723.50
PA + G25	2	1	51.6	10,529.23	1718.63
		2	48.4	13,964.85	2543.19

Data represent the probabilistic deconvolution of cellular fluorescence intensity into distinct subpopulations. The optimal number of components (K) for each experimental condition was determined by minimizing the BIC. Normal glucose (G5), high glucose (G25), palmitic acid (PA), and the combination (PA + G25). Parameters include the mixing proportion (%), the inverse-transformed mean fluorescence intensity (μ), and the standard deviation (σ). *n* = 600 cells.

**Table 4:** Quantitative parameters and phenotypic distribution of VEGFAR2-expressing subpopulations identified by Gaussian Mixture Model (GMM) analysis at 48 h.

Condition	Optimal K	Sub-Pop ID	Proportion (%)	Mean Int. Flu	Sta. Dev
G5	3	1	60.8	10,243.72	1431.68
		2	36.2	12,869.24	1799.96
		3	2.9	18,977.98	4193.37
G25	2	1	49.0	8988.24	1660.35
		2	50.9	14,981.13	2583.55
PA	6	1	29.8	8217.15	1001.36
		2	15.9	11,821.35	1602.77
		3	17.7	23,084.71	4136.81
		4	17.4	35,365.47	4026.87
		5	12.7	48,259.16	4528.25
		6	6.3	62,045.83	5488.14
PA + G25	3	1	69.1	18,755.93	2012.56
		2	30.3	23,920.06	3242.18
		3	0.5	39,734.05	3031.52

Data represent the probabilistic deconvolution of cellular fluorescence intensity into distinct subpopulations. The optimal number of components (K) for each experimental condition was determined by minimizing the BIC. Normal glucose (G5), high glucose (G25), palmitic acid (PA), and the combination (PA + G25). Parameters include the mixing proportion (%), the inverse-transformed mean fluorescence intensity (μ), and the standard deviation (σ). *n* = 600 cells.

## Data Availability

The datasets generated during and/or analyzed during the current study are available from the corresponding author on reasonable request.

## Supplementary Materials

The following supporting information can be downloaded at https://www.mdpi.com/article/10.3390/ijms27115144/s1.

## Abbreviations

The following abbreviations are used in this manuscript:

DM	Diabetes mellitus
DR	Diabetic retinopathy
FFAs	Free fatty acids
G25	High glucose
GMM	Gaussian mixture models
HDL	High-density lipoprotein
HIF1α	Hypoxia inducible factor 1 subunit alpha
iRBR	inner blood-retinal barrier
MGCs	Müller Glial Cells
NPDR	Non-proliferative diabetic retinopathy
PA	Palmitic acid
PA + G25	Palmitic acid more high glucose
PBS	Phosphate-buffered solution
PDR	Proliferative diabetic retinopathy
RECs	Retinal endothelial cells
Sp1	Specificity protein 1
T2DM	Type 2 diabetes
VEGFA	Vascular endothelial growth factor-A
VEGFR 1-2	VEGF Receptor 1 and 2
