# Supplementary figures and images for: Beyond Glucose: Palmitic Acid Influences VEGFA-VEGFR2 Angiogenic Signaling in Müller Glial Cells
Source: Int J Mol Sci. 2026 Jun 5;27(11):5144. doi: 10.3390/ijms27115144 (PMC13258240; doi:10.3390/ijms27115144)

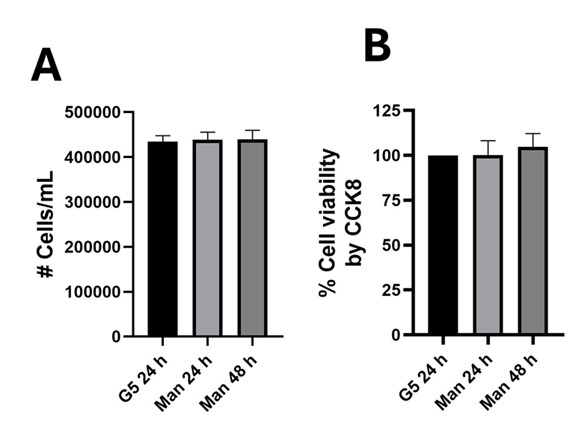

Supplement: Supplementary file 1 [file ijms-27-05144-s001.zip › Figure S1.jpg]

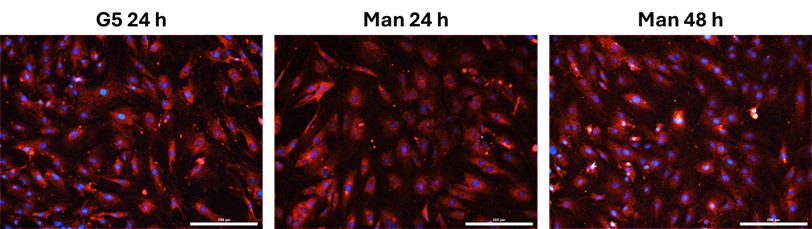

Supplement: Supplementary file 1 [file ijms-27-05144-s001.zip › Figure S2..jpg]

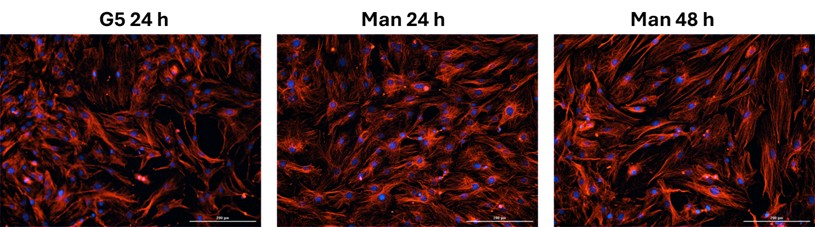

Supplement: Supplementary file 1 [file ijms-27-05144-s001.zip › Figure S3.jpg]

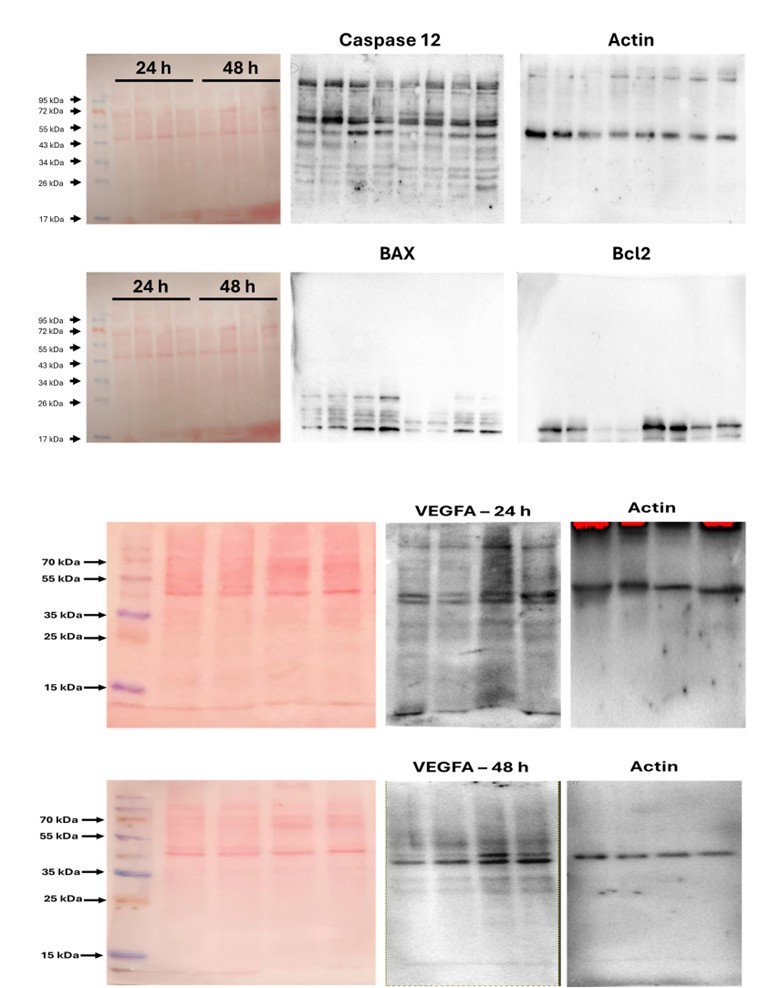

Supplement: Supplementary file 1 [file ijms-27-05144-s001.zip › Figure S4..jpg]
